# Association of Prolonged Nocturnal Hypoxemia with Clinical Worsening in Patients with Chronic Thromboembolic Pulmonary Hypertension Undergoing Pulmonary Endarterectomy

**DOI:** 10.31083/j.rcm2408240

**Published:** 2023-08-18

**Authors:** Hang Xu, Wu Song, Shanshan Zheng, Yige Huyan, Jiexu Ma, Zhaoji Zhong, Sheng Liu

**Affiliations:** ^1^Department of Cardiovascular Surgery, Key Laboratory of Pulmonary Vascular Medicine, Fuwai Hospital, National Clinical Research Center for Cardiovascular Diseases, National Center for Cardiovascular Diseases, Chinese Academy of Medical Sciences and Peking Union Medical College, 100037 Beijing, China

**Keywords:** sleep apnea, pulmonary hypertension, clinical worsening, hypoxemia

## Abstract

**Background:**

Obstructive sleep apnea (OSA) is common in patients with chronic thromboembolic pulmonary hypertension (CTEPH), but the pathological determinants of adverse outcomes remain unknown. This study aimed to investigate the prognostic significance of various sleep parameters in patients with CTEPH undergoing pulmonary endarterectomy.

**Methods:**

Consecutive patients diagnosed with CTEPH who underwent overnight cardiorespiratory polygraphy for the assessment of OSA were enrolled. Time-to-event analysis was performed investigating cardiorespiratory indices (e.g., apnea-hypopnea index [AHI], time percentage with oxygen saturation below <90% [T90]) and clinical worsening using the *log*-rank test, and multivariable Cox proportional hazard models adjusted for multiple confounders.

**Results:**

Of the 71 patients with operable CTEPH who underwent overnight cardiorespiratory polygraphy, 36 (50.7%) had OSA (AHI of ≥5) and 32 (45.1%) had nocturnal hypoxemia (T90 of ≥30%). A 10% increase in T90 was associated with a 27% greater risk of worse hemodynamics, as quantified by mean pulmonary artery pressure of ≥46 mmHg (odds ratio: 1.27, 95% confidence interval [CI]: 1.07–1.50, *p* = 0.006). Clinical worsening (CW) was experienced by 19 (26.8%) patients over a median follow-up of 26.8 months. AHI did not predict a higher risk of CW (hazard ratio [HR]: 1.00, 95% CI: 0.93–1.06, *p* = 0.906). A higher cumulative incidence of CW was seen in patients with nocturnal hypoxemia than in those with normoxemia (43.8% *vs*. 12.8%, *log*-rank *p* = 0.017). Cox regression analysis revealed the association between nocturnal hypoxemia and an increased risk of CW (HR: 3.27, 95% CI: 1.17–9.13, *p* = 0.024), and these associations persisted after covariate adjustment.

**Conclusions:**

Nocturnal hypoxemia quantified by T90 was a risk predictor of short- and long-term CW events among patients with operable CTEPH.

## 1. Introduction

Chronic thromboembolic pulmonary hypertension (CTEPH) is a rare yet life-threatening disease characterized by the chronic obstruction of major pulmonary arteries and microvasculature, causing a high incidence of morbidity and mortality [1, 2]. Pulmonary endarterectomy (PEA) is the preferred and most effective treatment course for patients with operable CTEPH. Emerging evidence supports the significant use of pulmonary hemodynamics as a unique resource and an important criterion for stratifying patients into low- and high-risk groups for in-hospital mortality [3]. A preoperative mean pulmonary arterial pressure (mPAP) of >46 mmHg is considered the standard to differentiate the high-risk from the low-risk groups for in-hospital mortality and postoperative complications [4]. Hence, perioperative risk stratification of patients with CTEPH provides prognostic implications for those undergoing PEA [1, 5].

Obstructive sleep apnea (OSA) is a common sleep-breathing disorder characterized by intermittent upper airway collapse or obstruction during sleep [6]. It affects an estimated 23.6% of the adult population globally, causing significant health and socioeconomic stress [7]. OSA is associated with several cardiovascular comorbidities and an increased risk of mortality [8]. OSA prevalence in patients with CTPEH is reported to be nearly 80% [9]. However, data regarding the role of OSA in terms of perioperative risk assessment and long-term prognostic implications in patients with CTEPH after PEA are limited. Specifically, the association between nocturnal sleep parameters and hemodynamic status (an alternative to in-hospital mortality) and long-term adverse outcomes in patients with operable CTEPH remains to be elucidated.

Therefore, this study aimed to, (1) explore the associations between various sleep parameters and pulmonary hemodynamics, as well as (2) investigate the impact of OSA parameters on the incidence of long-term adverse outcomes in patients with CTEPH undergoing PEA.

## 2. Material and Methods

### 2.1 Study Participants

This study retrospectively included consecutively diagnosed patients with CTEPH, who underwent PEA treatment from December 2014 to February 2022. CTEPH diagnoses were established based on pulmonary hypertension detected by right heart catheterization (RHC), the presence of mismatched perfusion defects on ventilation/perfusion scan, and evidence of thromboembolic disease on computed tomography pulmonary angiogram or conventional pulmonary angiography in patients who received at least 3 months of anticoagulant therapy [10]. Patients who exhibited risk factors for OSA, including nocturnal snoring, daytime sleepiness, obesity, enlarged neck circumference, or micrognathia, were advised to undergo overnight cardiorespiratory polygraphy (PG) to assess the presence and severity of sleep apnea. Final enrollment included patients with operable CTEPH undergoing PEA with nocturnal PG testing. This study excluded patients aged <18 years, those with insufficient or incomplete sleep data (<4 recorded hours), those with central sleep apnea, and those with hemodynamic instability or life-threatening cardiac arrhythmias. The study protocol was approved by the Ethics Committee of our institute, and written informed consent was obtained from all participants.

### 2.2 Baseline Clinical Data

Patient characteristics, including age, sex, body mass index (BMI), and pertinent medical histories, such as deep venous thrombosis (DVT) or acute pulmonary embolism (APE), were meticulously documented. Exercise capacity was evaluated through the recording of the six-minute walk distance (6MWD) and the World Health Organization-functional class (WHO-FC). Crucial laboratory findings, such as D-dimers and N-terminal pro-B-type natriuretic peptide (NT-proBNP), were thoroughly examined. Additionally, commercially available equipment was used for transthoracic echocardiography, and relevant parameters, such as left ventricular ejection fraction and tricuspid annular plane systolic excursion, were recorded.

### 2.3 Nocturnal Respiratory Events Study

All eligible patients with CTEPH underwent overnight cardiorespiratory PG monitoring within a week of admission after achieving clinical stabilization and before PEA. The primary parameters monitored included fingertip oxygen saturation (${\mathrm{SpO}}_{2}$), nasal airflow, and thoracoabdominal movements. The American Academy of Sleep Medicine guidelines were followed to evaluate and score nocturnal respiratory events, including apnea and hypopnea, which were considered significant if they lasted for at least 10 s and were accompanied by an airflow reduction of at least 90% or a peak signal excursion drop of 30% from the pre-event baseline, respectively [11]. The apnea-hypopnea index (AHI), the percentage of recording time with ${\mathrm{SpO}}_{2}$ of <90% (T90), and the oxygen desaturation index (ODI) were used to assess hypoxemia. T90 of ≥30% was considered a nocturnal hypoxemia indicator [12, 13]. Other hypoxia-related parameters evaluated included the mean ${\mathrm{SpO}}_{2}$, the minimum ${\mathrm{SpO}}_{2}$, the mean hypopnea time (HT), and apnea time (AT). An experienced technician, who was blinded to patients’ other clinical characteristics, scored the respiratory events.

### 2.4 Pulmonary Hemodynamics Assessment

RHC was performed to record pulmonary hemodynamics, with detailed previously described protocols [14]. Selected representative parameters from preoperative RHC included mPAP, pulmonary vascular resistance (PVR), and cardiac index. A board-certified cardiologist with extensive experience who was blinded to patient’s sleep study results performed RHC. A preoperative mPAP of >46 mmHg has been shown to increase the probability of in-hospital mortality in patients with operable CTEPH undergoing PEA [4]. Accordingly, patients were classified into two groups based on their hemodynamics: the worse hemodynamics group with mPAP ≥46 mmHg, which indicates a potentially higher risk of in-hospital mortality, and the better hemodynamics group with mPAP <46 mmHg, which suggests a potentially lower risk of in-hospital mortality, although no instances of in-hospital mortality were recorded.

### 2.5 Outcome and Follow-Up

A retrospective assessment was conducted on patients to determine the incidence of clinical worsening (CW) events. The CW was a composite endpoint of clinical worsening events, encompassing all-cause mortality, rehospitalization for heart failure, and residual pulmonary hypertension, which was defined as postoperative mPAP of >25 mmHg on the day of a repeat catheterization in need of further treatment such as postoperative targeted therapy or additional balloon pulmonary angioplasty (BPA) after PEA. The time to CW events was calculated from the date of the sleep study to the first CW occurrence or the end of the follow-up period. These events were identified through clinic visits, medical records, or telephone calls by research personnel who were blinded to the patient’s sleep study results.

### 2.6 Statistical Analysis

Continuous variables were presented as a mean ± SD or median (interquartile range) while categorical variables were shown as counts or percentages. An unpaired *t*-test was utilized for normally distributed, continuous variables for comparison of the baseline characteristics between two groups (worse hemodynamics* vs*. better hemodynamics; and CW *vs*. non-CW groups), and a nonparametric Kruskal-Wallis test was adopted for the non-normally distributed continuous variables. The *chi*-square test was used to compare categorical variables. Univariable and multivariable logistic regression analyses were used to investigate the relationship between sleep parameters and CTEPH patients with worse hemodynamics. *Log*-rank tests with Kaplan-Meier curves were used for time-to-event analysis that examines sleep parameters and long-term CW events, and Cox proportional hazard models adjusted for statistically or clinically relevant covariates.* p*-values of <0.05 were considered statistically significant. The statistical software programs *R* (*R version R* 4.1.1, R Foundation for Statistical Computing, Vienna, Austria) and GraphPad Prism 9 (GraphPad Software, San Diego, CA, USA) were used for all analyses.

## 3. Results

### 3.1 Study Population and Baseline Characteristics

This study initially enrolled 124 adult patients newly diagnosed with CTEPH. Among them, 92 patients with suspected OSA underwent overnight PG monitoring before undergoing PEA. This study excluded 17 patients with CTEPH with incomplete or inadequate sleep data due to hemodynamic instability or overt cardiac arrhythmia, as well as 4 patients with central sleep apnea. Finally, the analysis included 71 patients with operable CTEPH with successful PG results.

As shown in Table 1, 40% (29/71) of the study participants, who were aged 48.3 ± 12.7 years old and 67.6% male, were identified as having unfavorable hemodynamics. All participants received anticoagulants, and there were no significant differences in the usage of targeted medications between the preoperative poor hemodynamics cohort (mPAP ≥46 mmHg) and superior hemodynamics cohort (mPAP <46 mmHg). A history of DVT was more common in the poor hemodynamics cohort compared to the superior hemodynamics cohort (69.0% *vs*. 35.7%, *p* = 0.006). Moreover, patients in the poor hemodynamics cohort had higher mPAP (51.8 *vs*. 42.5 mmHg, *p* < 0.001), PVR levels (854.6 *vs*. 582.7 dyn⋅s⋅${\mathrm{cm}}^{-5}$, *p* = 0.006), and lower diurnal ${\mathrm{SpO}}_{2}$ levels (93.2 *vs*. 95.3%, *p* = 0.003). Of all patients with CTEPH, 36 (50.7%) suffered from OSA as determined by AHI of ≥5 events/h and the prevalence of nocturnal hypoxemia was 45.1% (32/71). Notably, the prevalence of nocturnal hypoxemia was significantly higher in the poor hemodynamics cohort when compared to the superior hemodynamics cohort (86.2% *vs*. 16.7%, *p* < 0.001). Additionally, the minimal ${\mathrm{SpO}}_{2}$ and mean ${\mathrm{SpO}}_{2}$ were significantly lower in the poor hemodynamics cohort when compared to the superior hemodynamics cohort (77.6% ± 8.4% *vs*. 81.9% ± 8.1%, *p* = 0.039 and 87.9% ± 3.5% *vs*. 92.0% ± 3.1%, *p* < 0.001, respectively). Finally, no significant differences were found between the cohorts regarding other sleep parameters, including AHI, longest AT, longest HT, mean AT, and mean HT.

**Table 1.** **Comparison of clinical characteristics of CTEPH patients undergoing PEA based on preoperative hemodynamic status**.

Variables		Poor hemodynamics	Superior hemodynamics	All (n = 71)	*p*
		mPAP ≥46 mmHg (n = 29)	mPAP <46 mmHg (n = 42)		
Age, years		49.4 ± 10.9	47.5 ± 13.9	48.3 ± 12.7	0.538
Male, n (%)		15 (51.7)	33 (78.6)	48 (67.6)	0.017
BMI, kg/$m^{2}$		24.3 ± 4.1	23.5 ± 4.8	23.9 ± 4.5	0.480
DVT history, n (%)		20 (69)	15 (35.7)	35 (49.3)	0.006
APE history, n (%)		8 (27.6)	5 (11.9)	13 (18.3)	0.093
6MWD (m)		378.2 ± 69.1	402.4 ± 114.6	390.6 ± 94.7	0.445
WHO-FC, III–IV, n (%)		20 (69)	25 (59.5)	45 (63.4)	0.417
${\mathrm{Targeted medications}}^{a}$, n (%)		12 (41.4)	17 (40.5)	29 (40.8)	0.939
Riociguat, n (%)		9 (31)	12 (28.6)	21 (29.6)	0.823
D-Dimer (ng/mL)		0.3 (0.2, 0.6)	0.4 (0.3, 0.9)	0.4 (0.2, 0.7)	0.078
NT-proBNP (mg/dL)		591.8 (190.0, 1574.0)	353.0 (143.1, 756.8)	405.5 (160.9, 1030.0)	0.103
LVEF (%)		67.7 ± 6.0	67.2 ± 5.9	67.4 ± 5.9	0.737
TAPSE (mm)		17.3 ± 3.5	18.0 ± 3.3	17.7 ± 3.4	0.421
Preoperative RHC					
	mPAP (mm Hg)	51.8 ± 11.7	42.5 ± 10.7	46.3 ± 12.0	<0.001
	CI (L/min/$m^{2}$)	2.5 ± 0.8	2.6 ± 0.6	2.5 ± 0.7	0.463
	${\mathrm{PVR (dyn\cdot s\cdot cm}}^{-\,5}$)	854.6 (588.8, 1238.0)	582.7 (459.6, 779.4)	654.4 (478.2, 962.6)	0.006
	Diurnal ${\mathrm{SpO}}_{2}$ (%)	93.2 ± 3.9	95.3 ± 4.0	94.4 ± 4.0	0.033
Preoperative sleep parameters					
	AHI (events/h)	7.4 (3.6, 13.1)	4.6 (2.0, 8.7)	5.1 (2.1, 11.7)	0.252
	ODI (events/h)	9.0 (6.0, 14.0)	5.2 (2.9, 9.6)	6.7 (4.0, 13.2)	0.016
	T90 (%)	55.5 (35.2, 82.0)	1.1 (0.2, 11.4)	19.0 (0.5, 55.8)	<0.001
	Nocturnal hypoxemia, n (%)	25 (86.2)	7 (16.7)	32 (45.1)	<0.001
	${\mathrm{minSpO}}_{2}$, %	77.6 ± 8.4	81.9 ± 8.1	80.1 ± 8.5	0.039
	Mean ${\mathrm{SpO}}_{2}$, %	87.9 ± 3.5	92.0 ± 3.1	90.3 ± 3.8	<0.001
	Longest AT, s	20.0 (11.6, 32.5)	20.5 (12.4, 31.7)	20.0 (12.0, 32.5)	0.710
	Longest HT, s	54.0 (42.0, 74.3)	61.2 (35.8, 81.0)	55.0 (37.0, 80.4)	0.490
	Mean AT, s	13.3 ± 6.1	15.2 ± 8.1	14.2 ± 7.2	0.327
	Mean HT, s	23.1 ± 9.9	27.3 ± 11.6	25.2 ± 10.9	0.142

Values are expressed as mean ± SD or mean (interquartile range). ^a^Including: phosphodiesterase-5 inhibitors, endothelin receptor antagonists, or prostanoids. Abbreviations: n, number of patients; CTEPH, chronic thromboembolic pulmonary hypertension; PEA, pulmonary endarterectomy; 6MWD, 6-minute walk distance; AHI, apnea-hypopnea index; APE, acute pulmonary embolism; AT, apnea time; BMI, body mass index; CI, cardiac index; DVT, deep venous thrombosis; HT, hypopnea time; LVEF, left ventricular ejection fraction; ${\mathrm{minSpO}}_{2}$, minimal ${\mathrm{SpO}}_{2}$; mPAP, mean pulmonary artery pressure; NT-proBNP, N-terminal pro-B-type natriuretic peptide; ODI, oxygen desaturation index; PVR, pulmonary vascular resistance; RHC, right heart catheterization; ${\mathrm{SpO}}_{2}$, oxygen saturation detected by pulse oximetry; TAPSE, tricuspid annular plane systolic excursion; T90, time percentage spent with ${\mathrm{SpO}}_{2}$ below 90%; WHO-FC, World Health Organization-functional class.

### 3.2 Associations of Sleep Parameters and Worse Hemodynamics

We investigated the associations between different sleep parameters and poor hemodynamics, aiming to identify key factors associated with potentially higher in-hospital mortality among CTEPH patients stratified by perioperative mPAP (≥46 mmHg) (Table 2). Logistic regression models were used to scale the independent variable T90 according to a 10-unit increment. This study revealed that a 10% increase in T90 was associated with a nearly 27% greater risk of being classified as having poor hemodynamics (odds ratio [OR]: 1.27, 95% confidence interval [CI]: 1.07–1.50, *p* = 0.006). T90 per 10-unit continued to carry a significantly increased risk in individuals with poor hemodynamics, even after adjusting for confounding factors such as age, sex, BMI, and anticoagulants (adjusted OR: 1.34, 95% CI: 1.08–1.68, *p* = 0.009). Notably, the conventional metrics of OSA severity, such as AHI, were not correlated with the high-risk group (OR: 0.94, 95% CI: 0.86–1.02; *p* = 0.142).

**Table 2.** **Associations of various sleep parameters with CTEPH patients with poor hemodynamics (perioperative mPAP ≥46 mmHg)**.

${\mathrm{Sleep parameters}}^{a}$	Unadjusted			${\mathrm{Model 1}}^{b}$			${\mathrm{Model 2}}^{c}$		
	OR	95% CI	*p*	OR	95% CI	*p*	OR	95% CI	*p*
AHI	0.94	(0.86–1.02)	0.142	0.93	(0.84–1.03)	0.144	0.93	(0.84–1.02)	0.136
ODI	0.98	(0.94–1.03)	0.518	0.98	(0.93–1.04)	0.504	0.98	(0.92–1.04)	0.457
T90 per 10-unit increment	1.27	(1.07–1.50)	0.006	1.22	(1.02–1.46)	0.026	1.34	(1.08–1.68)	0.009
Mean ${\mathrm{SpO}}_{2}$	0.75	(0.62–0.90)	0.002	0.77	(0.63–0.95)	0.013	0.76	(0.61–0.94)	0.010
${\mathrm{MinSpO}}_{2}$	0.99	(0.93–1.05)	0.757	1.01	(0.94–1.08)	0.844	1.01	(0.94–1.08)	0.829
Longest AT	0.98	(0.95–1.01)	0.243	0.99	(0.95–1.02)	0.463	0.99	(0.95–1.02)	0.455
Longest HT	0.99	(0.97–1.01)	0.336	0.99	(0.97–1.01)	0.261	0.99	(0.96–1.01)	0.211
Mean AT	0.98	(0.91–1.06)	0.581	0.99	(0.90–1.09)	0.899	0.99	(0.90–1.09)	0.903
Mean HT	0.98	(0.93–1.04)	0.550	0.98	(0.92–1.04)	0.471	0.98	(0.92–1.04)	0.424

^a^Each line represents a separate model. ^b^Model 1 adjusted for age, sex, and BMI. ^c^Model 2 adjusted for age, sex, BMI, and anticoagulant medication. CTEPH, chronic thromboembolic pulmonary hypertension; OR, odds ratio; AHI, apnea-hypopnea index; AT, apnea time; BMI, body mass index; CI, confidence interval; HT, hypopnea time; mPAP, mean pulmonary artery pressure; ODI, oxygen desaturation index; ${\mathrm{SpO}}_{2}$, oxygen saturation detected by pulse oximetry; T90, time percentage spent with ${\mathrm{SpO}}_{2}$ below 90%.

### 3.3 Association of Sleep Parameters and Long-Term Adverse Outcomes

**Fig. 1.**
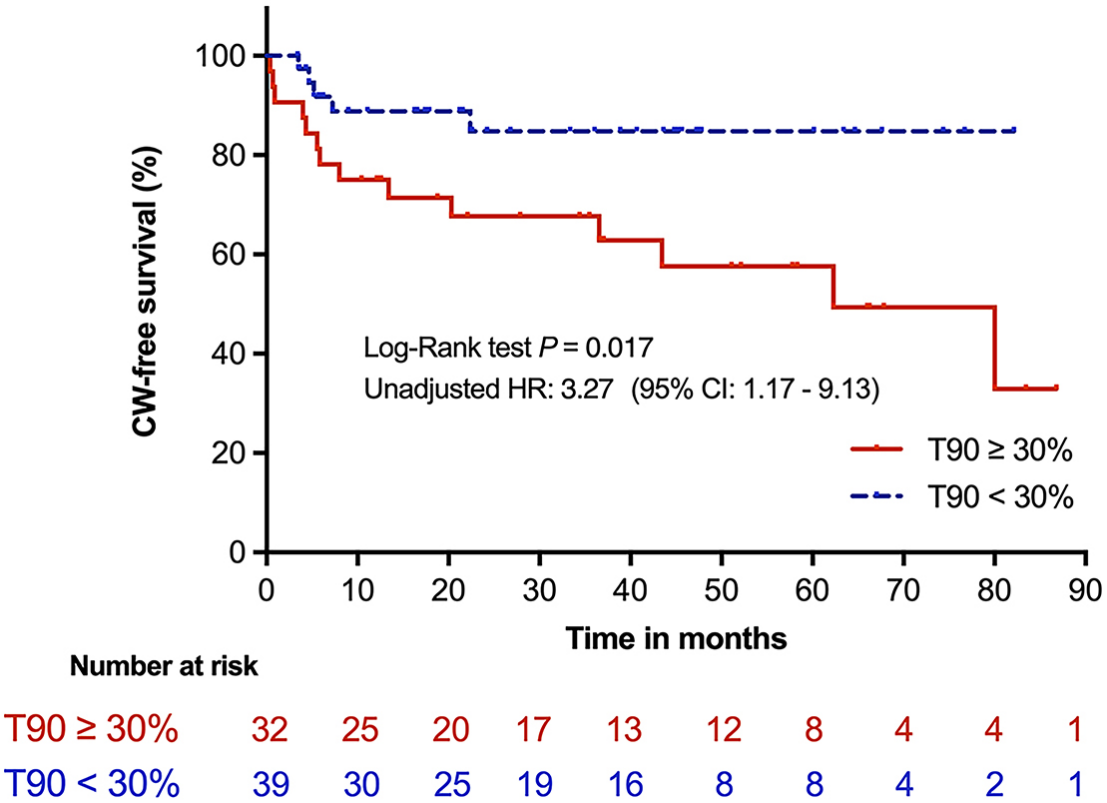
**Kaplan–Meier survival analysis of participants stratified by T90**. CW, clinical worsening; T90, time percentage with ${\mathrm{SpO}}_{2}$ below 90%; ${\mathrm{SpO}}_{2}$, oxygen saturation detected by pulse oximetry; HR, hazard ratio.

The incidence of CW events was 26.8% during a median follow-up period of 26.8 months (n = 19/71, Table 3). Patients who suffered from CW events were more likely to be female (53.6% *vs*. 25.0%, *p* = 0.028), have elevated levels of T90 (47.0% *vs*. 9.4%, *p* = 0.021), and have a higher percentage of nocturnal hypoxemia (73.7% *vs*. 34.6%, *p* = 0.003); there was no difference in AHI levels in patients without CW. Patients with nocturnal hypoxemia had a higher cumulative incidence of CW compared with patients with normoxemia (43.8% *vs*. 12.8%, *log*-rank *p* = 0.017, Fig. 1).

**Table 3.** **Comparisons between the CW and non-CW groups in patients with CTEPH undergoing PEA**.

Variables		CW (n = 19)	Non-CW (n = 52)	*p*
Age, years		51.0 ± 10.6	47.3 ± 13.4	0.280
Male, n (%)		9 (47.4)	39 (75.0)	0.028
BMI, kg/$m^{2}$		23.9 ± 3.3	23.8 ± 4.9	0.950
DVT history, n (%)		9 (47.4)	26 (50)	0.844
APE history, n (%)		6 (31.6)	7 (13.5)	0.095
6MWD (m)		408.7 ± 56.9	384.8 ± 104.2	0.518
WHO-FC, III–IV, n (%)		12 (63.2)	33 (63.5)	0.981
${\mathrm{Targeted medication}}^{a}$, n (%)		7 (36.8)	22 (42.3)	0.678
Riociguat, n (%)		7 (36.8)	14 (26.9)	0.418
D-Dimer (ng/mL)		0.5 (0.3, 1.4)	0.4 (0.2, 0.6)	0.114
NT-proBNP (mg/dL)		579.0 (242.3, 1396.0)	353.0 (153.4, 838.0)	0.215
LVEF (%)		67.3 ± 5.1	67.4 ± 6.3	0.980
TAPSE (mm)		16.6 ± 4.3	18.1 ± 2.9	0.113
Preoperative RHC				
	mPAP (mm Hg)	48.4 ± 12.9	45.5 ± 11.7	0.376
	CI (L/min/$m^{2}$)	2.7 ± 0.7	2.5 ± 0.6	0.211
	PVR ${\mathrm{(dyn\cdot s\cdot cm}}^{-\,5}$)	754.7 (559.1, 1006.0)	630.6 (458.4, 902.2)	0.479
	Diurnal ${\mathrm{SpO}}_{2}$ (%)	93.0 ± 4.2	94.9 ± 3.9	0.074
Preoperative sleep parameters				
	AHI (events/h)	4.7 (2.4, 12.7)	5.2 (2.1, 10.7)	0.979
	ODI (events/h)	7.1 (4.5, 13.2)	6.6 (3.6, 13.0)	0.546
	T90 (%)	47.0 (19.9, 79.0)	9.4 (0.5, 46.1)	0.021
	Nocturnal hypoxemia, n (%)	14 (73.7)	18 (34.6)	0.003
	${\mathrm{minSpO}}_{2}$, %	77.7 ± 9.7	81.0 ± 7.9	0.160
	Mean ${\mathrm{SpO}}_{2}$, %	88.4 ± 4.5	91.0 ± 3.4	0.013
	Longest AT, s	22.0 (11.6, 32.9)	19.4 (12.6, 31.7)	0.706
	Longest HT, s	54.5 (30.6, 81.5)	55.0 (42.1, 79.2)	0.841
	Mean AT, s	13.2 ± 6.0	14.7 ± 7.6	0.479
	Mean HT, s	25.4 ± 11.2	25.1 ± 11.0	0.919

Values are expressed as mean ± SD or mean (interquartile range). ^a^Including: phosphodiesterase-5 inhibitors, endothelin receptor antagonists, or prostanoids. n is the number of participants. Abbreviations: CTEPH, chronic thromboembolic pulmonary hypertension; PEA, pulmonary endarterectomy; 6MWD, 6-minute walk distance; AHI, apnea-hypopnea index; APE, acute pulmonary embolism; AT, apnea time; BMI, body mass index; CI, cardiac index; CW, clinical worsening; DVT, deep venous thrombosis; HT, hypopnea time; LVEF, left ventricular ejection fraction; ${\mathrm{minSpO}}_{2}$, minimal ${\mathrm{SpO}}_{2}$; mPAP, mean pulmonary artery pressure; NT-proBNP, N-terminal pro B-type natriuretic peptide; ODI, oxygen desaturation index; PVR, pulmonary vascular resistance; RHC, right heart catheterization; ${\mathrm{SpO}}_{2}$, oxygen saturation detected by pulse oximetry; TAPSE, tricuspid annular plane systolic excursion; T90, time percentage spent with ${\mathrm{SpO}}_{2}$ below 90%; WHO-FC, World Health Organization-functional class.

Univariable analysis (Table 4) correlated nocturnal hypoxemia (hazard ratio [HR]: 3.27, 95% CI: 1.17–9.13, *p* = 0.024), previous APE (HR: 3.79, 95% CI: 1.35–10.63, *p* = 0.011), and riociguat (HR: 3.03, 95% CI: 1.09–8.45, *p* = 0.034) with CW risk. However, other factors, including age, sex, BMI, 6MWD, WHO-FC, and other sleep parameters showed no significant association with CW events. Notably, there was no association between AHI and CW (HR: 1.00, 95% CI: 0.93–1.06, *p* = 0.906). Only a few covariates were included in the multivariable analysis due to the low number of reported CW events. Nocturnal hypoxemia remained a significant risk factor for CW with an adjusted HR of 2.95 (95% CI: 1.04–8.33, *p* = 0.040) despite adjusting for age and BMI. Similarly, after adjusting for APE history and riociguat usage, patients with nocturnal hypoxemia experienced an 80% increased CW risk (HR: 3.07, 95% CI: 1.09–8.67, *p *= 0.034).

**Table 4.** **Univariable Cox regression analysis of associations between risk factors and CW**.

Variables	HR	95% CI	*p*
Age	1.03	(1.00–1.08)	0.082
Female	2.12	(0.86–5.24)	0.102
BMI	1.01	(0.92–1.12)	0.788
DVT history	0.97	(0.39–2.38)	0.939
APE history	3.79	(1.35–10.63)	0.011
6MWD	1.00	(1.00–1.00)	0.584
WHO-FC, III–IV	0.91	(0.35–2.36)	0.852
${\mathrm{Targeted medication}}^{a}$	0.68	(0.27–1.75)	0.429
Riociguat	3.03	(1.09–8.45)	0.034
D-Dimer	1.49	(0.80–2.77)	0.207
NT-proBNP	1.00	(1.00–1.00)	0.898
LVEF	1.01	(0.94–1.09)	0.747
TAPSE	0.89	(0.76–1.04)	0.140
mPAP	1.02	(0.98–1.06)	0.401
CI	1.60	(0.78–3.25)	0.198
PVR	1.00	(1.00–1.00)	0.856
${\mathrm{SpO}}_{2}$	0.94	(0.86–1.04)	0.245
AHI	1.00	(0.93–1.06)	0.906
ODI	1.00	(0.96–1.04)	0.996
Nocturnal hypoxemia	3.27	(1.17–9.13)	0.024
${\mathrm{minSpO}}_{2}$	0.99	(0.95–1.03)	0.531
Mean ${\mathrm{SpO}}_{2}$	0.94	(0.85–1.03)	0.163
Longest AT	1.00	(0.98–1.03)	0.992
Longest HT	1.00	(0.99–1.02)	0.805
Mean AT	0.98	(0.92–1.06)	0.660
Mean HT	1.02	(0.98–1.06)	0.415

^a^Including: phosphodiesterase-5 inhibitors, endothelin receptor antagonists, or prostanoids. Abbreviations: 6MWD, 6-minute walk distance; AHI, apnea-hypopnea index; APE, acute pulmonary embolism; AT, apnea time; BMI, body mass index; CI, cardiac index; CW, clinical worsening; DVT, deep venous thrombosis; HT, hypopnea time; LVEF, left ventricular ejection fraction; ${\mathrm{minSpO}}_{2}$, minimal ${\mathrm{SpO}}_{2}$; mPAP, mean pulmonary artery pressure; NT-proBNP, N-terminal pro-B-type natriuretic peptide; ODI, oxygen desaturation index; PVR, pulmonary vascular resistance; ${\mathrm{SpO}}_{2}$, oxygen saturation detected by pulse oximetry; TAPSE, tricuspid annular plane systolic excursion; WHO-FC, World Health Organization-functional class; HR, hazard ratio.

## 4. Discussion

The present investigation revealed fresh insights into the correlation between hypoxemic burden, as measured by T90, and unfavorable outcomes in patients with CTEPH who were considered suitable candidates for PEA. Further, the prevalence of OSA in CTEPH patients with high clinical suspicion of sleep-disordered breathing was remarkable, and those who suffered from nocturnal hypoxemia exhibited worse hemodynamics as evaluated by preoperative mPAP. Patients with nocturnal hypoxemia had a nearly 3-fold increased susceptibility to CW events in a median follow-up of 26.8 months.

OSA has gained increasing attention in pre-capillary pulmonary hypertension as a potential risk factor for disease severity and poor prognosis in cardiovascular diseases. Our study revealed nearly half of the patients with operative CTEPH suffer from OSA, consistent with a reported prevalence ranging from 20% to 57% [15, 16, 17, 18]. Previous studies have revealed that OSA may contribute to postoperative complications in patients undergoing cardiac surgery, such as coronary artery bypass graft; however, relevant evidence was scarce in patients with operable CTEPH [19, 20]. We revealed that the duration of oxygen saturation below 90% as quantified by T90, contrary to AHI, may serve as a more independent factor for both short- and long-term adverse outcomes in patients with CTEPH after adjusting for covariates. Our findings partially agree with a recent multicenter trial, which suggested that moderate-to-severe sleep apnea measured by AHI did not pose additional cardiovascular risks in patients with acute coronary syndrome [21]. This inconsistency may be because AHI, which is the traditional metric for sleep apnea by counting the total number of respiratory events per sleep hour, fails to capture key aspects of sleep apnea, such as the hypoxemic sequelae.

The quantification of nocturnal hypoxemia using T90 is a promising method that provides more accurate disease severity and outcome indications in both healthy and pathological populations. Recent literature emphasizes that T90 is a superior prognostic indicator in patients with heart failure [22] and older community-dwelling males [8, 23, 24], compared to the widely accepted AHI. Furthermore, a prospective cohort study revealed T90 as an independent predictor of all-cause mortality in patients with chronic stable heart failure and reduced ejection fraction [25]. Similar associations between T90 and mortality were also observed in patients with advanced chronic kidney disease [22, 26]. T90 is an independent predictor of pulmonary vascular and right ventricular remodeling [14], as well as acute pulmonary embolic recurrence in patients with pulmonary vascular diseases. Consistent with this literature, our study supports the notion that prolonged hypoxemia is a reliable predictor of unfavorable outcomes in patients with CTEPH [27].

PEA stands as the definitive curative approach to CTEPH, offering symptomatic relief and a better prognosis to eligible candidates [28]. A patient is deemed operable when adequate surgically accessible thromboembolic material is present, and a proportionate PVR indicates the absence of extensive distal disease. Notably, highly specialized centers have shown optimal success rates [3], and our institution recorded an overall survival rate of 91.2% and 83.9% at 5 and 10 years, respectively, for patients with CTEPH who underwent PEA [29]. Our study revealed no patients that died during hospitalization after PEA. Prior studies have suggested poor hemodynamics are associated with greater peri-operative risk even though that finding was not confirmed in this cohort [4]. However, our long-term follow-up analysis revealed an unsatisfactory survival rate of 73.2%, highlighting the utmost importance of perioperative risk stratification. Our current findings further contribute to the existing knowledge by demonstrating the important role of OSA and nocturnal hypoxemia as significant risk factors for long-term outcomes in patients with operable CTEPH undergoing PEA.

Several pathological mechanisms underlie the association between CTEPH, OSA, and nocturnal hypoxemia. Studies suggest that OSA-induced intermittent hypoxia and systemic inflammation contribute to pulmonary vascular remodeling and vasoconstriction, thereby increasing pulmonary arterial pressure, which could be transient or persistent [16, 24]. Furthermore, an increase in intrathoracic negative pressure, venous return, right ventricular preload, and stroke volume could lead to elevated pulmonary artery blood flow and pressure during obstructive events. Operable CTEPH is characterized by proximal thrombotic obstructions unlike CTEPH, which primarily impacts distal vasculature that can be treated with BPA. A major element that promotes the development of pulmonary arterial thrombus is presumably the shearing stress experienced during dramatic thoracic swings in sleep apnea. Additionally, OSA can increase the afterload of the left ventricle by elevating transmural pressure [30]. Prolonged hypoxemia concomitant with this condition may further increase the formation of reactive oxygen species, which consequently accelerates the enlargement of proximal thrombi, necessitating surgical procedures to relieve the obstruction. However, further mechanistic studies are needed to establish causal links between these factors.

Our study demonstrated that T90 may represent a more dependable predictor of unfavorable outcomes in patients with operable CTEPH than AHI alone. Ideally, sleep parameters, particularly T90, should be evaluated preoperatively to enhance preoperative risk stratification and develop more precise treatment strategies for patients with CTEPH. Moreover, overnight oximetry may be appropriate for assessing hypoxemic burden in patients with CTEPH without OSA-related symptoms. Regrettably, in the present study, there was a lack of regular subsequent treatment for OSA among patients during hospitalization and follow-up. This may be attributed to the lack of recognition or diagnosis, prioritization of other interventions, incomplete assessment, complexity of comorbidities, lack of consensus on treatment approaches, and patient preferences and limitations. While the absence of interventions limits the assessment of the modifiability of abnormal sleep as a risk factor, it provides valuable insights into the natural course and impact of sleep-related abnormalities on clinical outcomes in CTEPH patients post-PEA. But presumably, nocturnal hypoxemia with oxygen supplementation therapy can be oxygen therapy or by the first-line therapy of OSA, which is continuous positive airway pressure. Long-term follow-up studies that evaluate the effectiveness of different interventions on outcomes and risk stratification in CTEPH patients with abnormal sleep can provide valuable insights into the modifiability of this risk factor and its influence on clinical trajectories.

The observed association between riociguat use and increased complications in our study may be attributed to several factors, including the limitations of our sample size, the presence of confounding variables, and potential patient selection bias. A small sample size and potential confounding factors should be considered when interpreting the results of the relationship between riociguat use and procedural complications in CTEPH therapies. Caution should also be exercised in extrapolating the findings to a broader population due to the small number of patients receiving riociguat and the potential influence of sampling variability on the statistical analysis. Furthermore, the retrospective nature of the study and the presence of confounding variables, such as disease severity and concomitant medications, make it challenging to establish a definitive causal relationship between riociguat use and increased complications. Moreover, patient selection bias, influenced by factors like physician preference and medication accessibility, may have introduced inherent bias in our results, highlighting the need for future studies with larger sample sizes, prospective designs, and rigorous control of confounders to provide a more definitive understanding of the association.

The multifaceted analysis of overnight cardiorespiratory metrics and thorough evaluation of the associations between T90 and short- and long-term outcomes are the strength of the study. However, several limitations warrant discussion. Firstly, the retrospective and single-center study design restricts the result’s generalizability. Secondly, the cross-sectional observational design used to evaluate short-term prognosis may not establish a causal relationship between T90 and in-hospital mortality risk. Thirdly, the small number of events may preclude a complete elimination of potential confounders, thereby limiting the study’s statistical power. Lastly, the portable monitoring device used to assess the presence and severity of OSA, although well-established and validated, may be subject to limitations [31].

## 5. Conclusions

Overnight hypoxemic burden quantified by T90 was an independent predictor of CW events in patients with CTEPH who were operable for PEA. Nocturnal hypoxemia investigation may aid in the risk stratification of CTEPH. The potential benefits of supplemental oxygen in reducing T90 and improving outcomes in patients with CTEPH should be further explored in prospective studies and randomized trials.

## Data Availability

The datasets used and/or analyzed during the current study are available from the corresponding author on reasonable request.
